# Supplementary material for: CTHRC1 Induces Pancreatic Stellate Cells (PSCs) into Myofibroblast-like Cancer-Associated Fibroblasts (myCAFs)
Source: Cancers (Basel). 2023 Jun 27;15(13):3370. doi: 10.3390/cancers15133370 (PMC10340658; doi:10.3390/cancers15133370)
Supplement: Supplementary file 1 [file cancers-15-03370-s001.zip › cancers-2362422-supplementary.pdf]

Supplementary Table S1. Primer lists of RT-qPCR

Target species	Gene	Forward primer (5′-3′)	Reverse primer (5′-3′)
Homo sapiens	POLR2A	TGAAAGTTTTGCGCTGTGTC	ACCCTCGCATATGTTTTTGC
Homo sapiens	GAPDH	CTTTGGTATCGTGGAAGGACTC	GTAGAGGCAGGGATGATGTTCT
Homo sapiens	FAP	GGCTCACGTGGGTTACTGAT	GTCTGCCAGTCTTCCCTGAA
Homo sapiens	SULF1	AAGTGACGGGTTCTTGGTTG	GGCAAGGTCAAATGAGGTGT
Homo sapiens	THBS2	TATTCCCGAGACCAACGAAG	ACATCATCGTCACTCCCACA
Homo sapiens	COL5A2	TGGATTGATCCTAACCAAGGA	GGTACACTGGATGGGTTTGC
Homo sapiens	COL10A1	TGCTGCCACAAATACCCTTT	TGGGTAGTGGGCCTTTTATG
Homo sapiens	COL11A1	TGGAACTTTCCCAGAAGACTTT	CTCCCAACCTCAACACCAAT
Homo sapiens	CTSK	CCGCAGTAATGACACCCTTT	GCACCCACAGAGCTAAAAGC
Homo sapiens	LUM	CCTGGGGCCATTATCCTACT	CGTTAGCAACACGTAGACATTCA
Homo sapiens	POSTN	CGGTCAGTCGGTATCCTGTT	GTCACCGTCACATCCTATCTCA
Homo sapiens	COL6A3	CGAAAGACGAAGGAACTTGC	TTGTTTTCGTTTCCACCACA
Homo sapiens	COL3A1	GATCAGGCCAGTGGAAATGT	GTGTGTTTCGTGCAACCATC
Homo sapiens	COL8A1	GAAAGCCAGGTTTCCTTGGT	TTCCTGGCTCTCCCTTAGGT
Homo sapiens	SPARC	GCTCCACCTGGACTACATCG	TACAGGGTGACCAGGACGTT
Homo sapiens	LOX	ACATTACCACAGTATGGATGAGTTT	TGTGCAGTACATGCAAATCG
Homo sapiens	MFAP5	TTGCACCTTCCACAGATGAC	TGGTGAAGCATAACTGATGAATG
Homo sapiens	VEGFA	AGGCCAGCACATAGGAGAGA	TTTCTTGCGCTTTCGTTTTT
Homo sapiens	PDGFA	GGAGTGAGGATTCTTTGGACAC	GTAAATGACCGTCCTGGTCTTG
Homo sapiens	EGF	GCCAAGCAGTCTGTGATTGA	CTGATGGCATAGCCCAATCT
Homo sapiens	HGF	GAGAGTTGGGTTCTTACTGCACG	CTCATCTCCTCTTCCGTGGACA
Homo sapiens	CTGF	TGACCTGGAAGAGAACATTAAGAAG	CACAGAATTTAGCTCGGTATGTCTT
Homo sapiens	FGF	CCGTCAAGGAAATACACCAGTT	TCAGCTCTTAGCAGACATTGGA
Homo sapiens	IL1B	TCTTCTTCGACACATGGGATAAC	CAGTTCATATGGACCAGACATCA
Homo sapiens	IL6	TCTGGATTCAATGAGGAGAC	GAACTGGATCAGGACTTTTG
Homo sapiens	IL8	GAGAGTGATTGAGAGTGGACCAC	CACAACCCTCTGCACCCAGTTT
Homo sapiens	IL10	GATTTCTTCCCTGTGAAAACAAGAG	CTTTAGCTTCGGGTCAATGC
Homo sapiens	TGFB1	AGCTGTACCAGAAATACAGCAACA	ACTCCGGTGACATCAAAAGATAAC
Homo sapiens	CXCL12	AGAGCCAACGTCAAGCATCT	CTTTAGCTTCGGGTCAATGC

Homo sapiens	GM-CSF	ACATGGTTTGACTCCCGAAC	GCCTGGCCCTAAAAAGAGTC
Homo sapiens	FGFR	GAACAGGCATGCAAGTGAGA	ACGAAGCACTGACCTCCCTA
Homo sapiens	ACTA2	TCCCTTGAGAAGAGTTACGA	GATGCTGTTGTAGGTGGTTT
Homo sapiens	THY1	GAAGGTCCTCTACTTATCCGCC	TGATGCCCTCACACTTGACCAG
Homo sapiens	TAGLN	TCCAGGTCTGGCTGAAGAAT	GCTCCATCTGCTTGAAGACC
Homo sapiens	CLEC3B	AGCTCAAGAGCCGTCTGGACAC	GGAAGGTCTTCGTCTGGGTGAA
Homo sapiens	COL14A1	GTGTCCGAGGAATGGTATAA	AGGATGGTGTTAATGTCAGC
Homo sapiens	CD74	AAGCCTGTGAGCAAGATGCGCA	AGCAGGTGCATCACATGGTCCT
Homo sapiens	SLPI	AGCGTGACTTGAAGTGTTGCATG	GAAAGGACCTGGACCACACAGA
Mus musculus	ACTA2	TGCTGACAGAGGCACCACTGAA	CAGTTGTACGTCCAGAGGCATAG
Mus musculus	THY1	CCTTACCCTAGCCAACTTCACC	TTATGCCGCCACACTTGACCAG
Mus musculus	TAGLN	GCAGATGGAACAGGTGGCTCAA	CCCAAAGCCATTAGAGTCCTCTG
Mus musculus	CLEC3B	CGAGGAACTCAAGAACAGGATGG	GCCTCATGGAAGGTCTTCGGTT
Mus musculus	COL14A1	GTCAGGCTTCAGTGATGCTCTG	ATTTGCCACCGAGCACACAAGC
Mus musculus	IL6	TACCACTTCACAAGTCGGAGGC	CTGCAAGTGCATCATCGTTGTTC
Mus musculus	CD74	GCTGGATGAAGCAGTGGCTCTT	GATGTGGCTGACTTCTTCCTGG
Mus musculus	SLPI	AAGCAGAGGTGCTGCCAAGATG	TCTGGCAGACATTGGGAGGGTT
Mus musculus	GM-CSF	GAGCAGTTGTGTGCCACCTA	AGCTGGCTTAGGCACTGTGT
Mus musculus	TGFB	TGCCCTCTACAACCAACACA	GTTGGACAACTGCTCCACCT
Mus musculus	IL1B	GCTGCTTCCAAACCTTTGAC	AGCTTCTCCACAGCCACAAT
Mus musculus	FGFR	GAGACAGACTGGTCTTAGGCAAA	CCAATCATTTTCATCATCTCCAT

Supplementary Table S2. Information of datasets included for comparison of CTHRC1 mRNA expression between paired tumor/normal, and between paired cancer/stroma samples.

Series Accession	Publication Year	Title	Country	Diagnosis	Sample Type	Number	mRNA Expression Platform	Publication PMID
GSE62452	2016	Microarray gene-expression profiles of 69 pancreatic tumors and 61 adjacent non-tumor tissue from patients with pancreatic ductal adenocarcinoma	USA	Pancreatic ductal adenocarcinoma	Paired tumor/normal	60/60	Affymetrix Human Gene 1.0 ST Array [transcript (gene) version]	27197190
					Unpaired tumor/normal	9/1		
GSE183795	2022	Microarray gene-expression profiles of 139 pancreatic tumor,102 adjacent non-tumor tissue from patients with pancreatic ductal adenocarcinoma and 3 normal pancreas from donors.	USA	Pancreatic ductal adenocarcinoma	Paired tumor/normal	100/100#	Affymetrix Human Gene 1.0 ST Array	36426859
					Unpaired tumor/normal/	39/2		
					Unclassified	3		
GSE15471	2009	Whole-Tissue Gene Expression Study of Pancreatic Ductal Adenocarcinoma	Romania	Pancreatic ductal adenocarcinoma	Paired tumor/normal	36/36&	Affymetrix U133 Plus 2.0 whole-genome chips	19260470, 28881803
					Unpaired tumor/normal	0/0		
GSE16515	2009	Expression data from Mayo Clinic Pancreatic Tumor and Normal samples	USA	Pancreatic ductal adenocarcinoma	Paired tumor/normal	16/16	Affymetrix Human Genome U133 Plus 2.0 Array	19732725, 27749787, 23936393
					Unpaired tumor/normal	20/0		
GSE93326	2018	Gene expression from laser capture microdissected pancreatic cancer epithelium and stroma	Germany	Pancreatic ductal adenocarcinoma	Paired tumor /stroma	66/66	Nugen Ovation RNA-Seq System V2	30658994, 29249692, 29440233
					Unpaired tumor /stroma	0/57		
GSE164665	2021	Transcriptomic Analysis of Laser Capture Microdissected Tumors of Pancreatic Ductal Adenocarcinoma	USA	Pancreatic ductal adenocarcinoma (All)	Paired cancer/stroma	19/19	Illumina NextSeq 500 (Homo sapiens)	-
					Unpaired cancer/stroma	0/0		

# 80 (40/40) paired tumor/normal samples were excluded from study due to sample overlapping with GSE62452.

& Three samples were tested in duplicates, resulting in 78 (39/39) paired tumor/normal data.

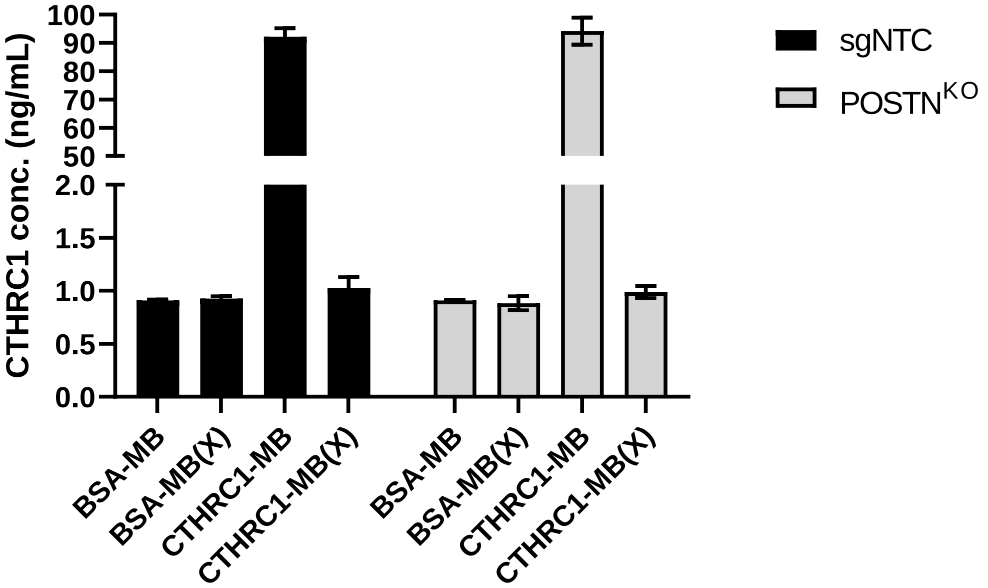

Supplementary Figure S1. CTHRC1 concentration in pancreatic stellate cell (PSC) conditioned media, determined by sandwich ELISA.

BSA-MB/CTHRC1-MB: Conditioned media of PSC with or without POSTN-knockout (sgNTC and PONTN^{KO}, respectively) treated by magnetic bead (MB)-conjugated BSA or CTHRC1.

BSA-MB(X)/CTHRC1-MB(X): Conditioned media of PSC with or without POSTN-knockout (sgNTC and PONTN^{KO}, respectively) treated by magnetic bead (MB)-conjugated BSA or CTHRC1, harvested after BSA and CTHRC1 removed by a magnetic stand.

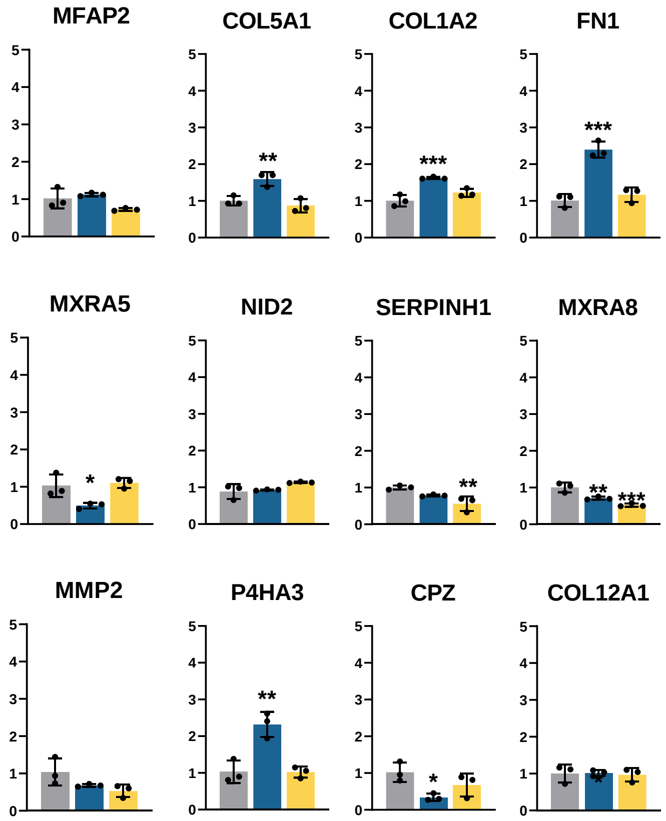

Supplementary Figure S2. Extracellular matrix (ECM)-related genes not upregulated by CTHRC1 in pancreatic stellate cells (PSC).

The expression of 35 ECM-related genes that showed high correlation with CTHRC1 (r > 0.8) was measured after PSC were treated with PBS, TGF-β (2.1 nM), or CTHRC1 (1.7 nM) for 24 hours. Except 15 genes that significantly showed upregulation, 18 of the 35 ECM-related genes did not show a significant change in expression, and two genes (SERPINH1 and MXRA8) showed a significantly reduced expression.

Figure 3A

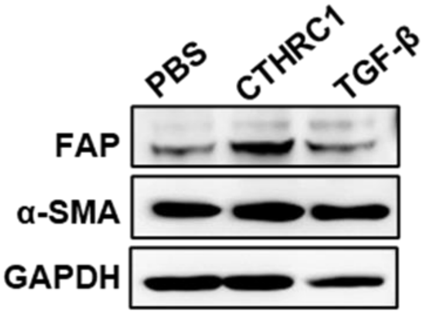

GAPDH

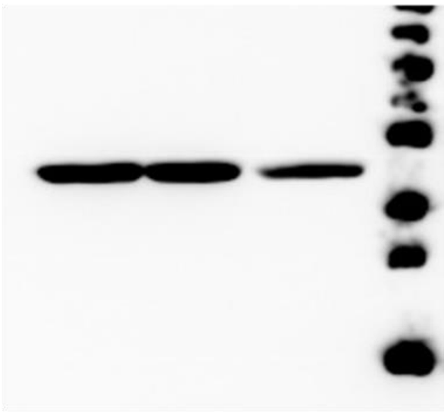

α-SMA

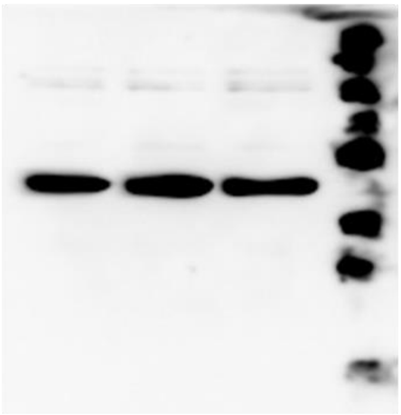

FAP

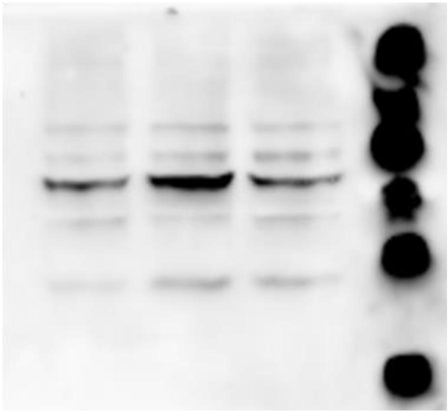

Figure 6B

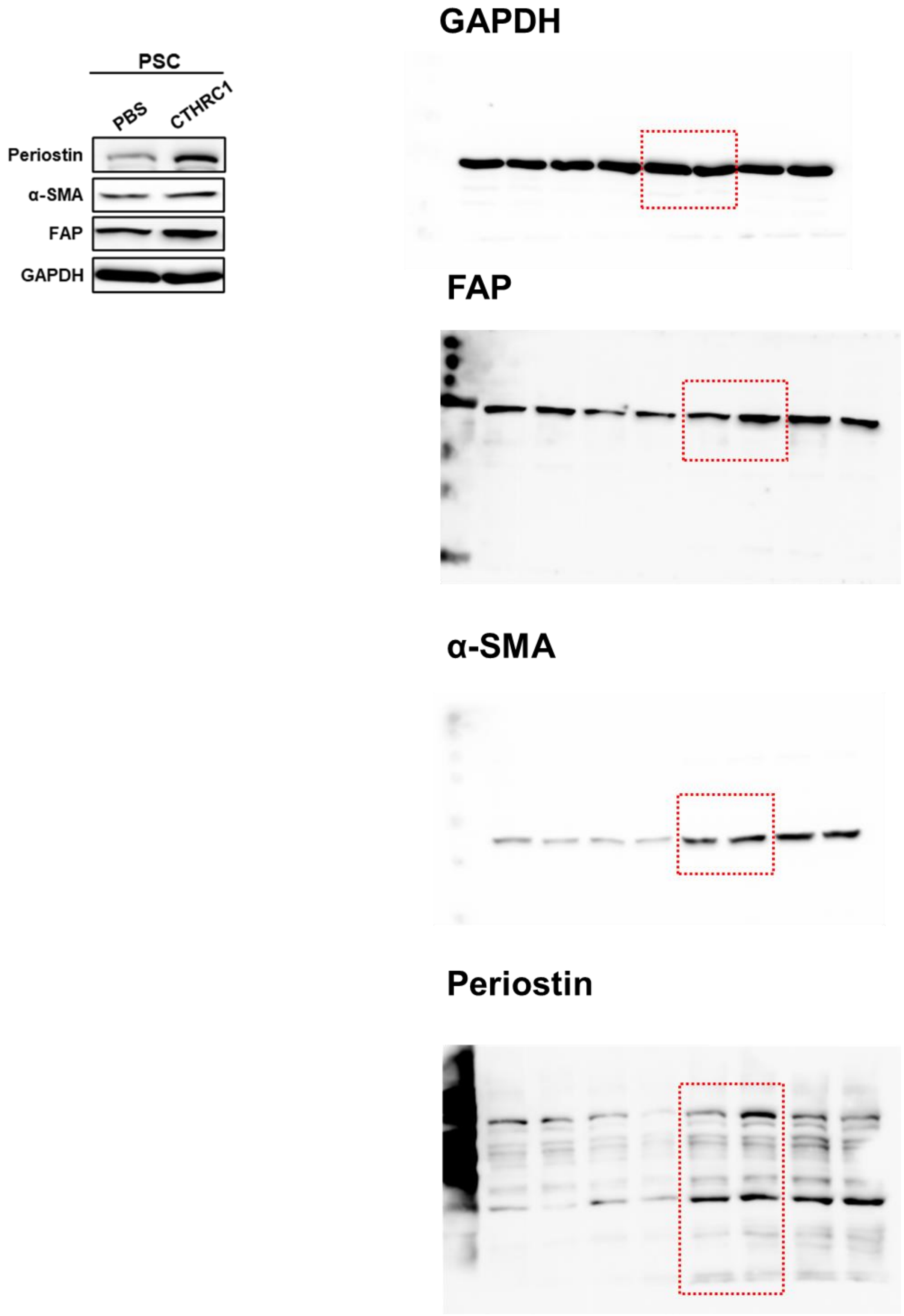

Supplementary Figure S3. Uncropped images of western blot membranes shown in Figure 3A and Figure 6B.

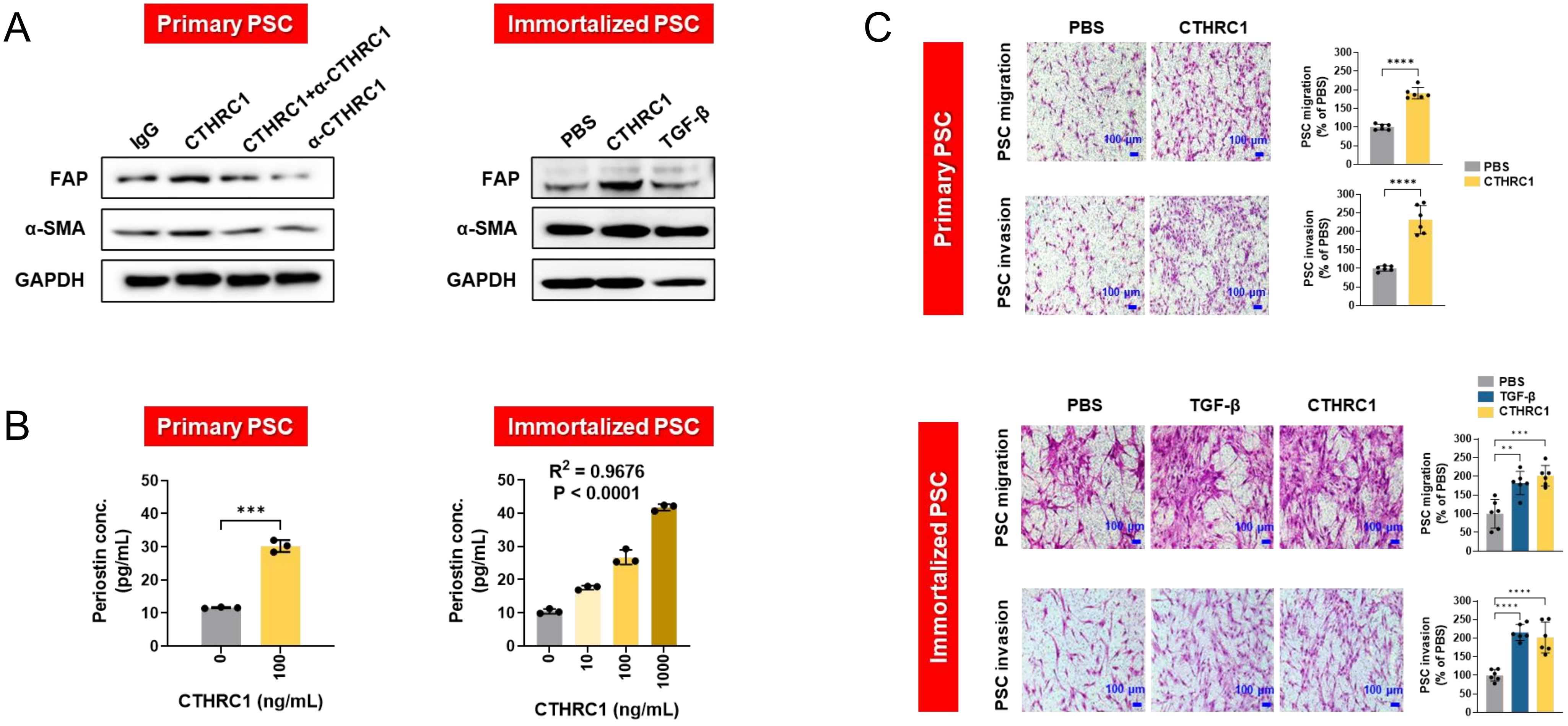

Supplementary Figure S4. In vitro studies conducted using primary pancreatic stellate cells (PSC) and immortalized PSC generated similar results.

(**A**) Activation of primary PSC and immortalized PSC after treatments with: (primary PSC) IgG, CTHRC1 (1.7 nM), CTHRC1+αCTHRC1 (anti-CTHRC1 mAb, 69.4 nM), or αCTHRC1, (immortalized PSC) PBS, CTHRC1 (1.7 nM), or TGF-β (2.1 nM) for 24 hours, demonstrated by protein expression of FAP and α-SMA using Western blot. (**B**) Concentration of periostin in primary PSC and immortalized PSC after treatment with: (primary PSC) 0 and 100 ng/mL CTHRC1, (immortalized PSC) 0, 10, 100, and 1000 ng/mL CTHRC1 for 24 hours, determined by sandwich ELISA. (**C**) Activation of metastatic capacity of primary PSC and immortalized PSC by the treatment of PBS, CTHRC1 (1.7 nM), or TGF-β (2.1 nM, only for immortalized PSC) for 24 hours.

PBS

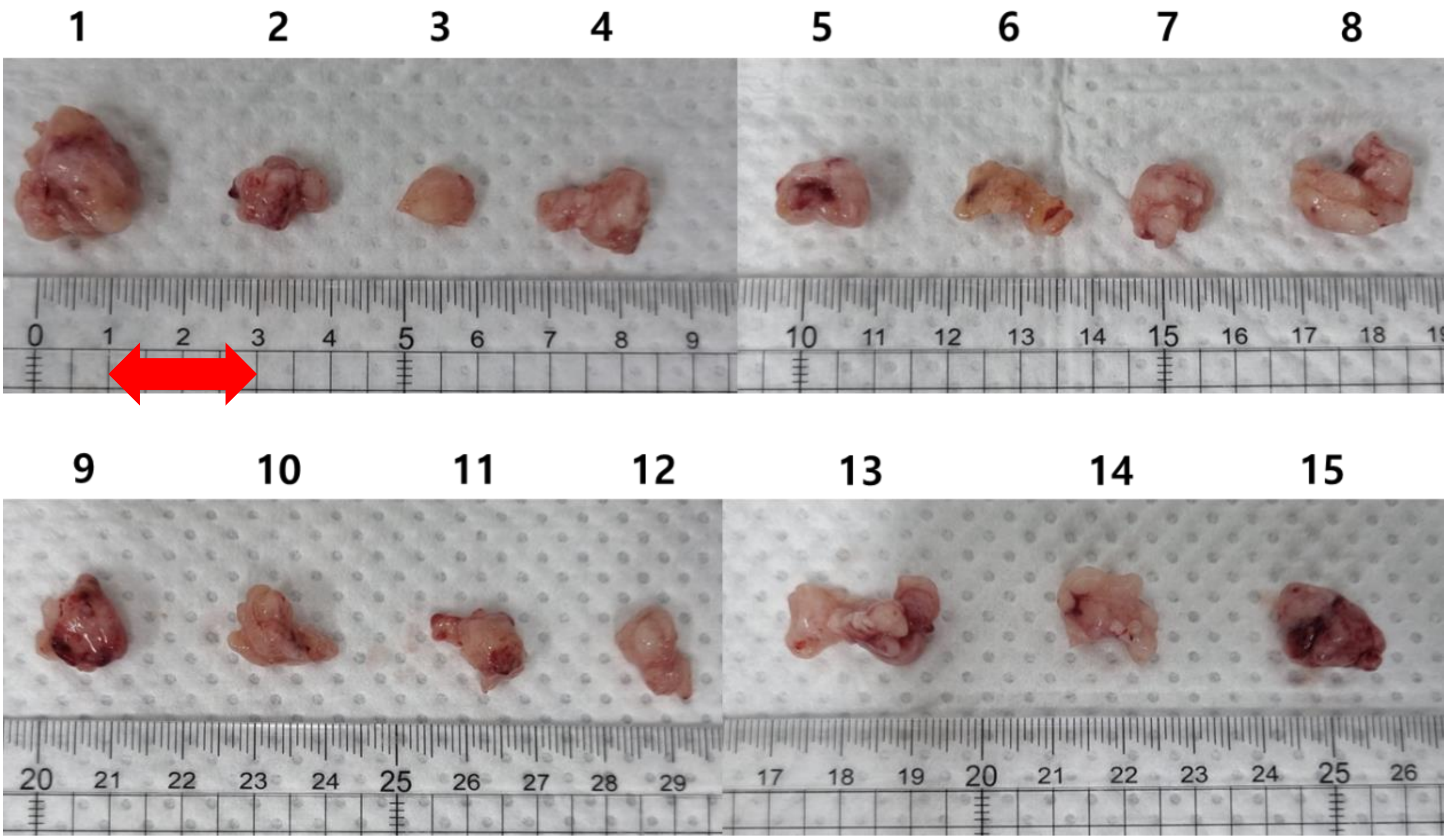

Gemcitabine

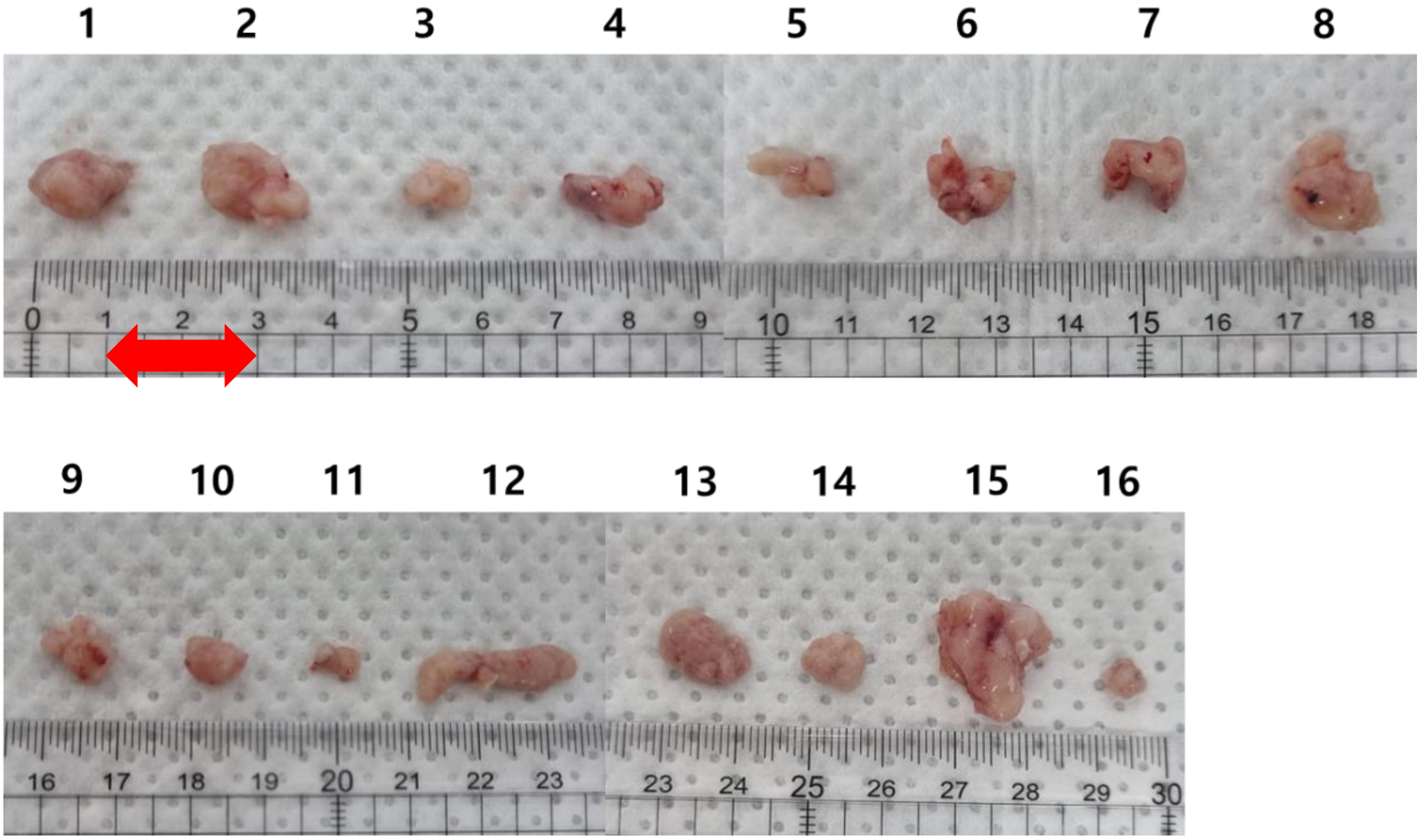

α-CTHRC1

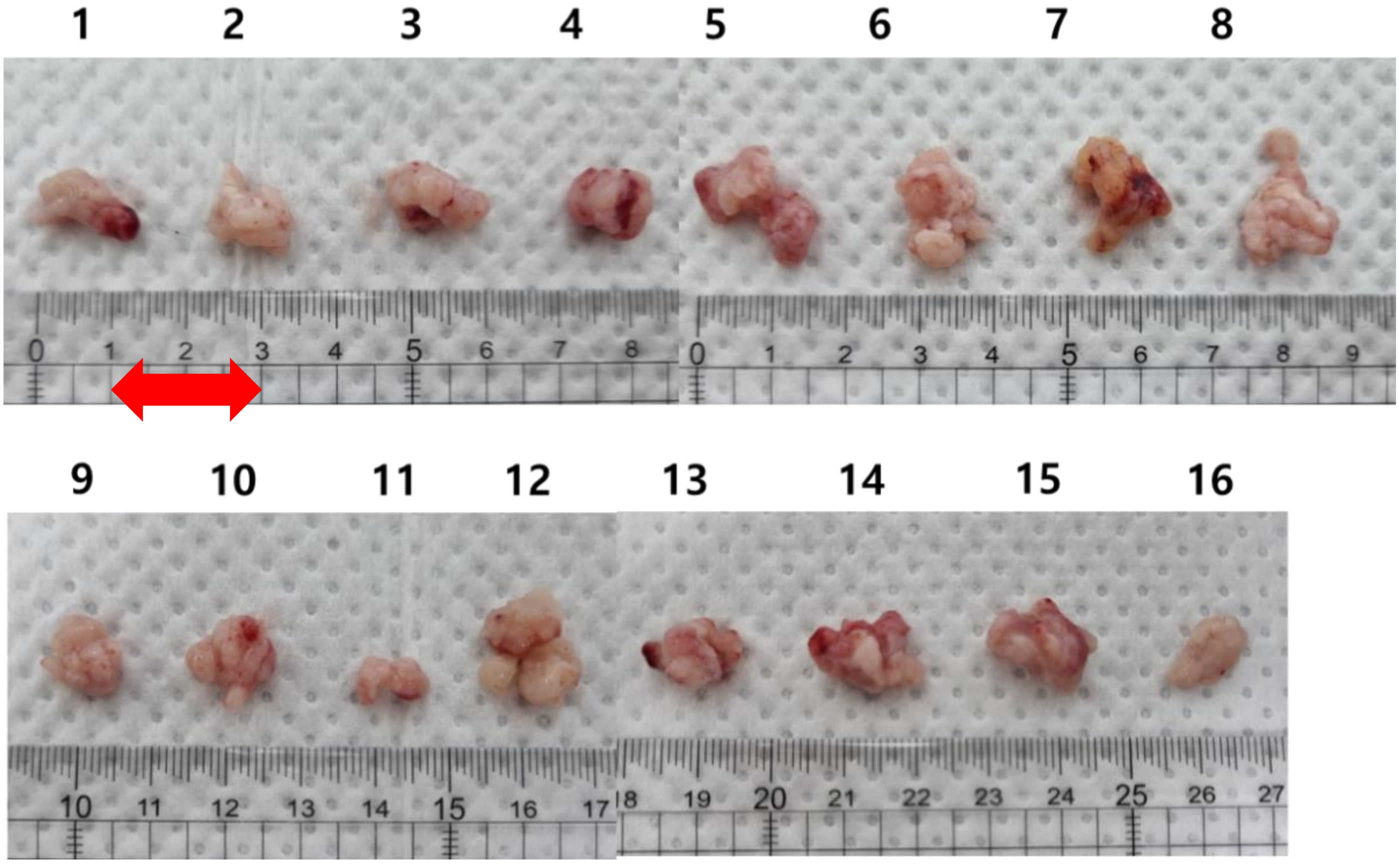

Gem + α-CTHRC1

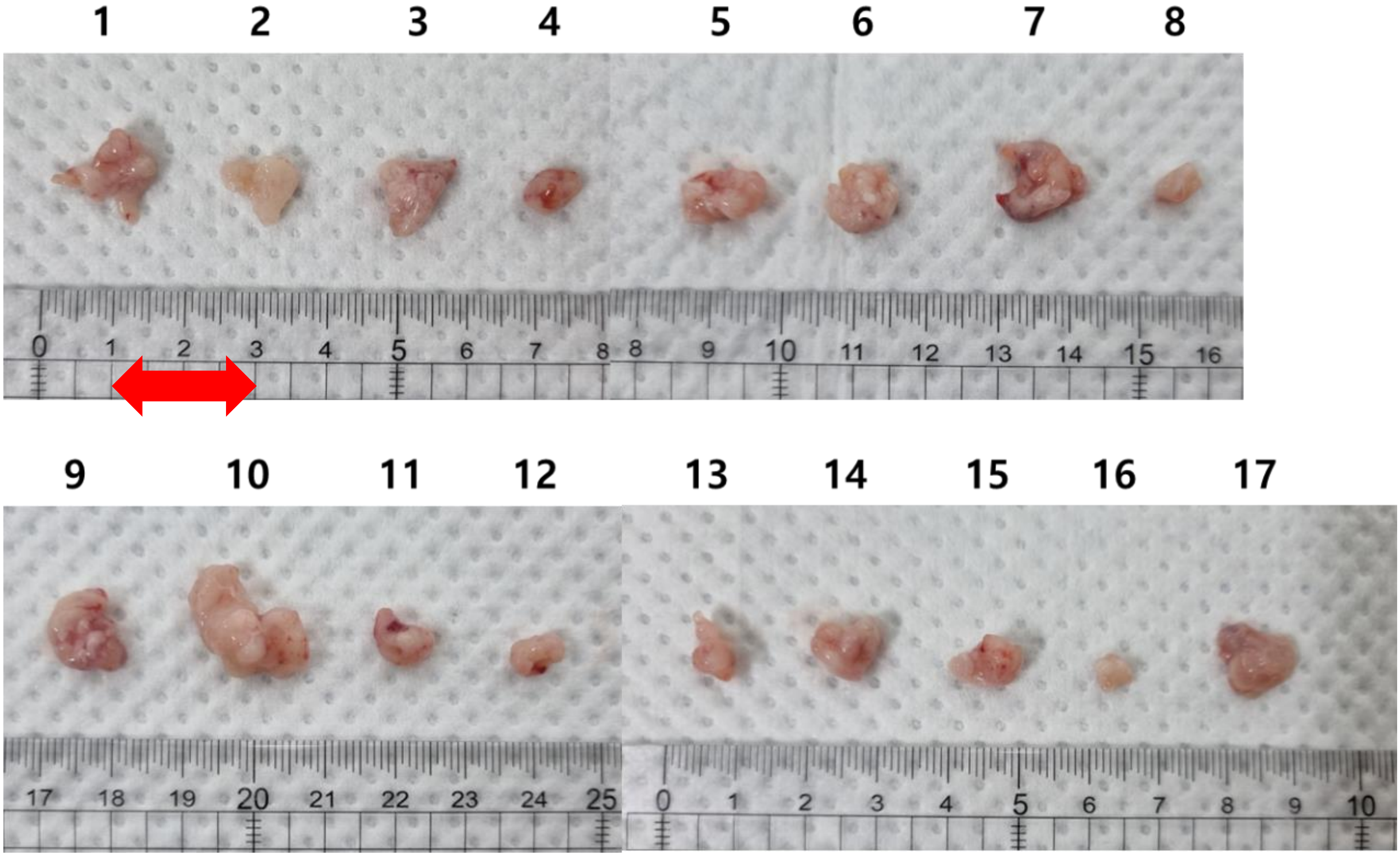

Supplementary Figure S5. Tumor images of CFPAC-1 orthotopic mouse model.

Tumor images were obtained after the sacrifice of the animals in the CFPAC-1 orthotopic mouse model. Red arrows (2 cm) are used to scale the images to 100% of the actual size.
